# Supplementary material for: Impacts of climate change on reproductive phenology in tropical rainforests of Southeast Asia
Source: Commun Biol. 2022 Apr 21;5:311. doi: 10.1038/s42003-022-03245-8 (PMC9023445; doi:10.1038/s42003-022-03245-8)
Supplement: Supplementary file 1 — Supplemental Material [file 42003_2022_3245_MOESM1_ESM.pdf]

Supplementary Information

Impacts of climate change on reproductive phenology in tropical rainforests of Southeast Asia

Authors: Shinya Numata[1*], Koharu Yamaguchi[2], Masaaki Shimizu[2], Gen Sakurai[3], Ayaka Morimoto[1], Noraliza Alias[4], Nashatul Zaimah Noor Azman[4], Tetsuro Hosaka[5], Akiko Satake[6*]

Affiliations:
[1] Department of Tourism Science, Tokyo Metropolitan University, Tokyo 192-0397, Japan.
[2] Graduate School of Systems Life Science, Kyushu University, Fukuoka 819-0395, Japan.
[3] National Institute for Agro-Environmental Sciences, NARO, Tsukuba 305-8604, Japan.
[4] Forest Research Institute Malaysia, 52109 Kepong, Selangor, Malaysia.
[5] Graduate School of Advanced Science and Engineering, Hiroshima University, Hiroshima 739-8529, Japan.
[6] Department of Biology, Faculty of Science, Kyushu University, Fukuoka 819-0395, Japan.

*Correspondence to:

Shinya Numata (nmt@tmu.ac.jp) and Akiko Satake (akiko.satake@kyudai.jp)

Supplementary Information:
Supplementary Figure 1– Supplementary Figure 11
Supplementary Table 1 and Supplementary Table 2

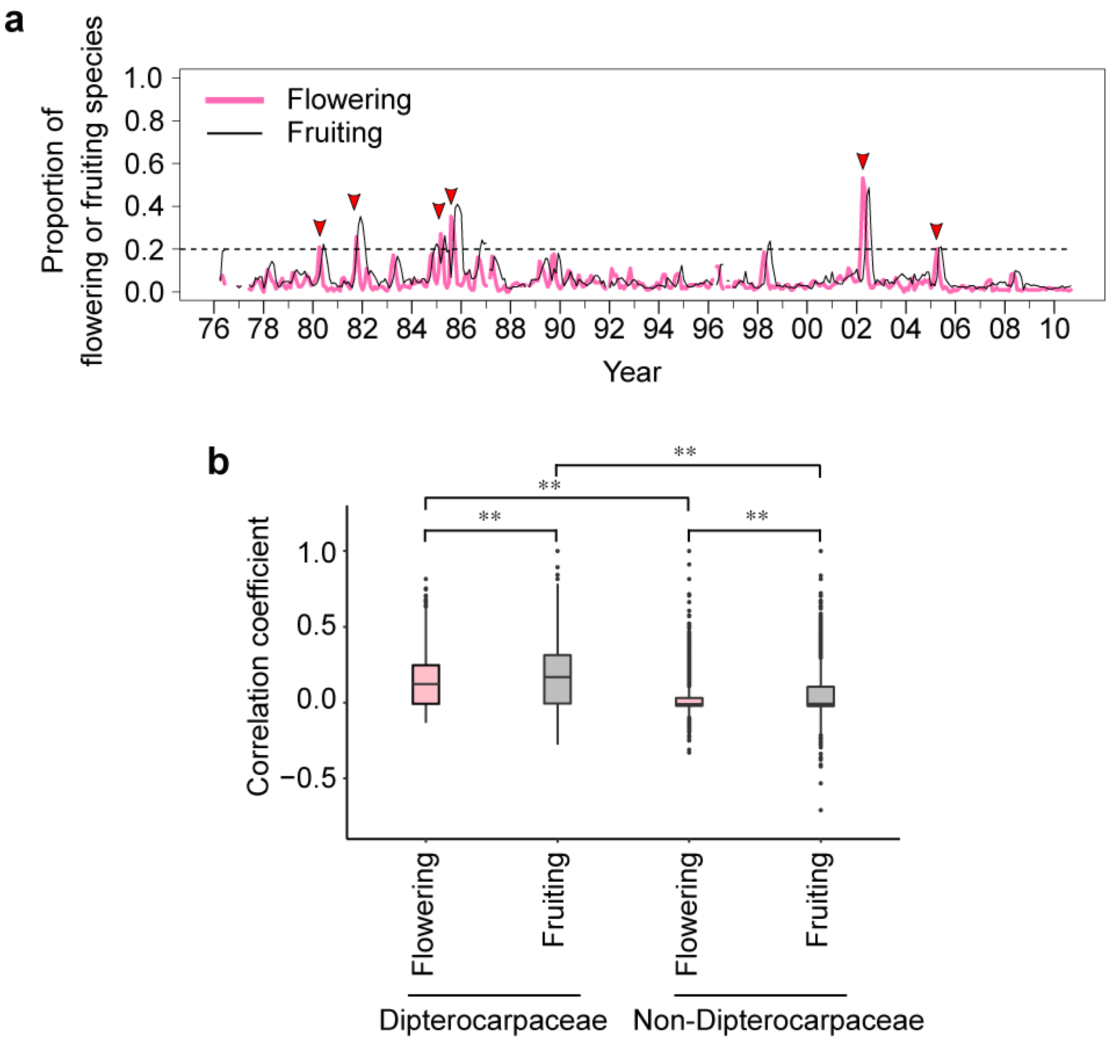

Supplementary Figure 1 | Proportion of flowering and fruiting species in 210 tropical tree species and correlation of flowering and fruiting phenology among species. a, The horizontal arrows indicate large flowering events with flowering of more than 20% of the monitored species. A dashed line represents the level of 20% of flowering or fruiting species. **b**, Mean Pearson's correlation coefficient values of flowering and fruiting binary data over all species pairs, calculated for Dipterocarpaceae (95 species) and non-Dipterocarpaceae species (115 species). Symbols indicate the results of the two-way ANOVA test. $^{**}P < 0.01$. When there were missing values for at least one species, the time point was removed.

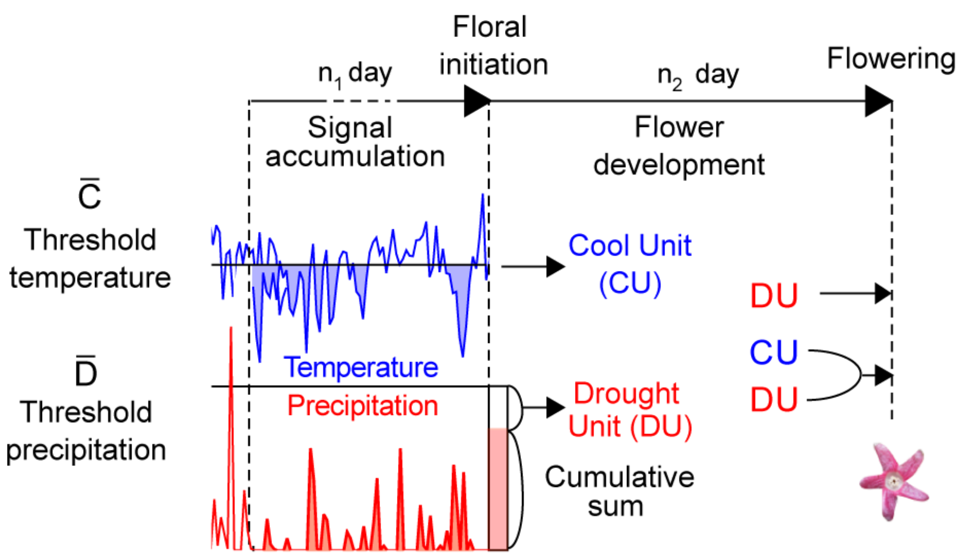

Supplementary Figure 2 | Model structure

The model includes the period of flower development n_2 days after the onset of floral initiation and the accumulation of environmental signals over n_1 days prior to the onset of floral initiation. The cool unit (CU) was equal to the cumulative sum of the negative differences between the threshold temperatures ($\bar{C}$) and the daily mean temperatures over n_1 days. The drought unit (DU) equalled the difference between the threshold precipitation level ($\bar{D}$) and the cumulative sum of precipitation over n_1 days. Two models were used to describe the relationship between the flowering probability and environmental cues. The two models involved the drought only (DU) and synergistic cold plus drought (CU × DU) models.

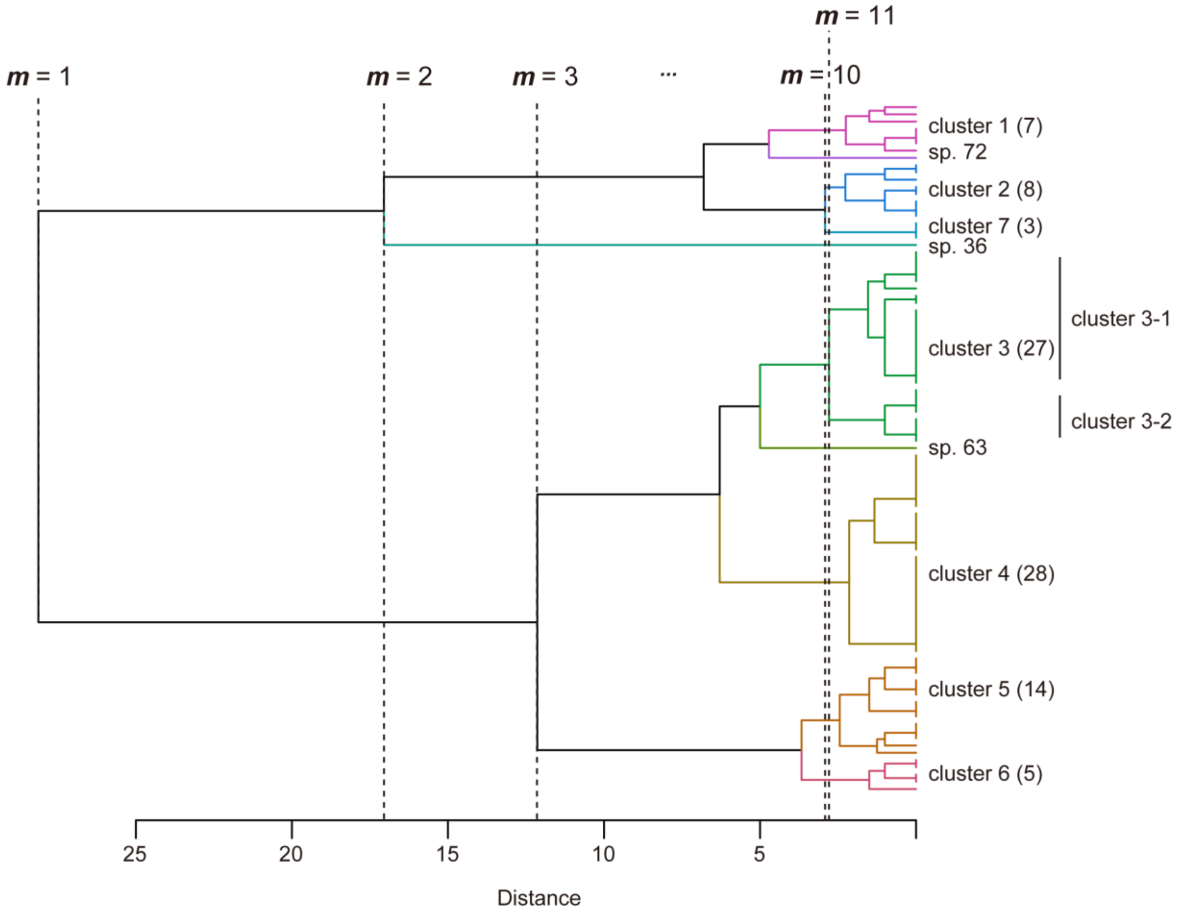

Supplementary Figure 3 | Illustration for the forward selection of the cluster number. A dendrogram indicates the results of the time-series clustering of 95 dipterocarp species. The value m is the number of phenological clusters examined for model fitting. The species grouped into the same cluster at $m = 10$ are illustrated by the same colour. The optimal cluster number ($\hat{m} = 10$) was identified according to the forward selection of optimal $\hat{m}$ values based on the minimization of the AIC. When $m = 11$, cluster 3 was divided into two subclusters (clusters 3-1 and 3-2). Clusters with fewer than four species (cluster 7 and independent species sp. 36, sp. 63 and sp. 72) were removed before phenology forecasting was conducted due to their small sample size.

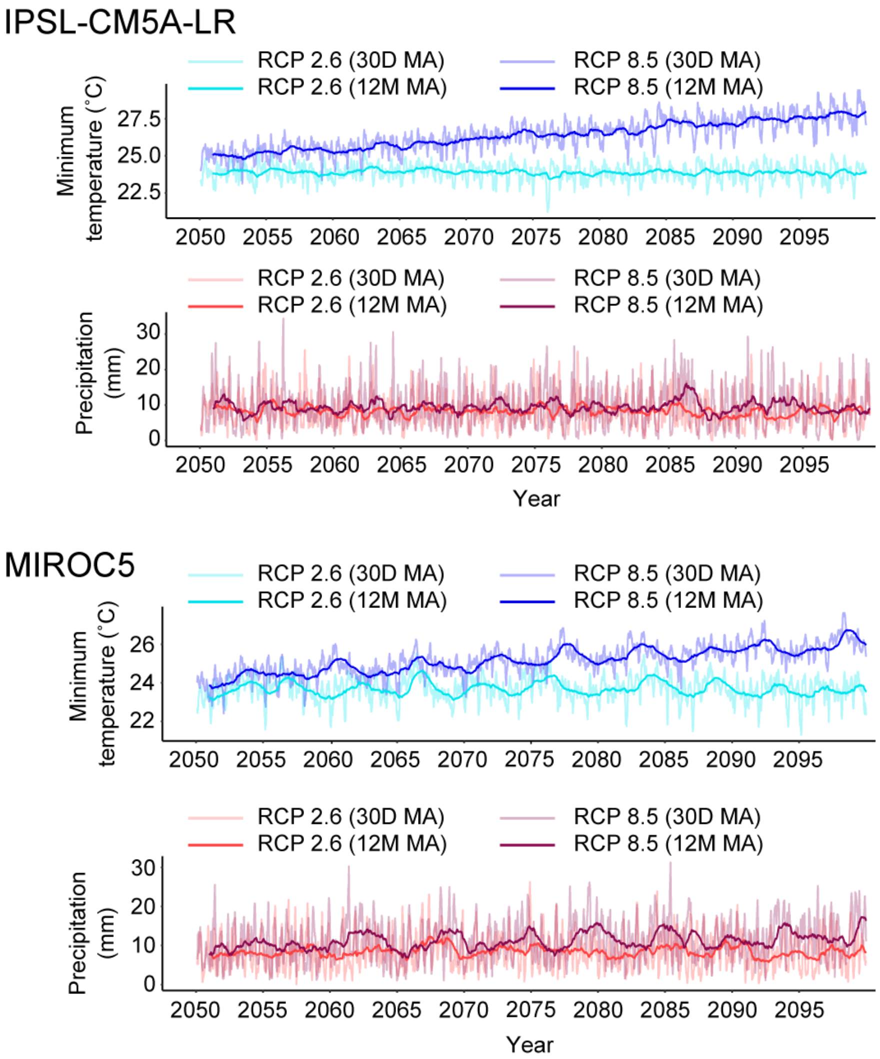

Supplementary Figure 4 | Future minimum temperature and precipitation (2050–2099) values simulated by PSL-CM5A-LR and MIROC5. Future climate conditions were simulated under two climate scenarios (RCP2.6 and RCP8.5). The 30-day (30 D) and 12-month (12 M) moving averages (MAs) were plotted.

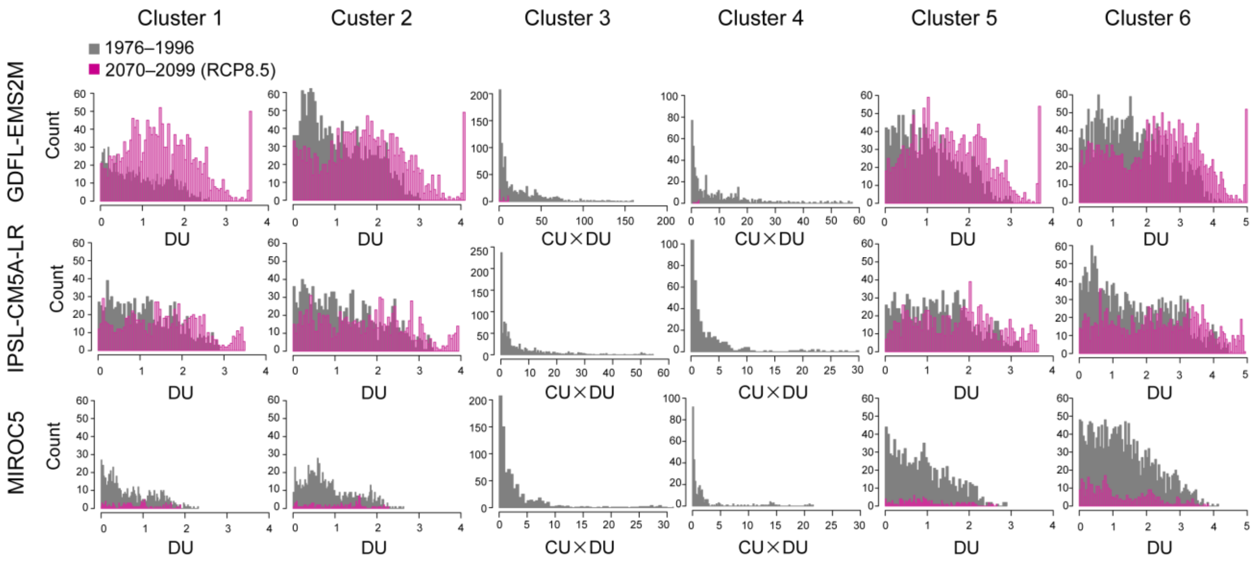

Supplementary Figure 5 | Predicted changes in flowering cues under the RCP8.5 scenario. The predicted environmental signals (predicted with DU or CU × DU) during 1976-1996 (grey) and 2070-2099 (pink) were plotted as histograms for each cluster and for each climate model (GDFL-EMS2M, IPSL-CM5A-LR, and MIROC5).

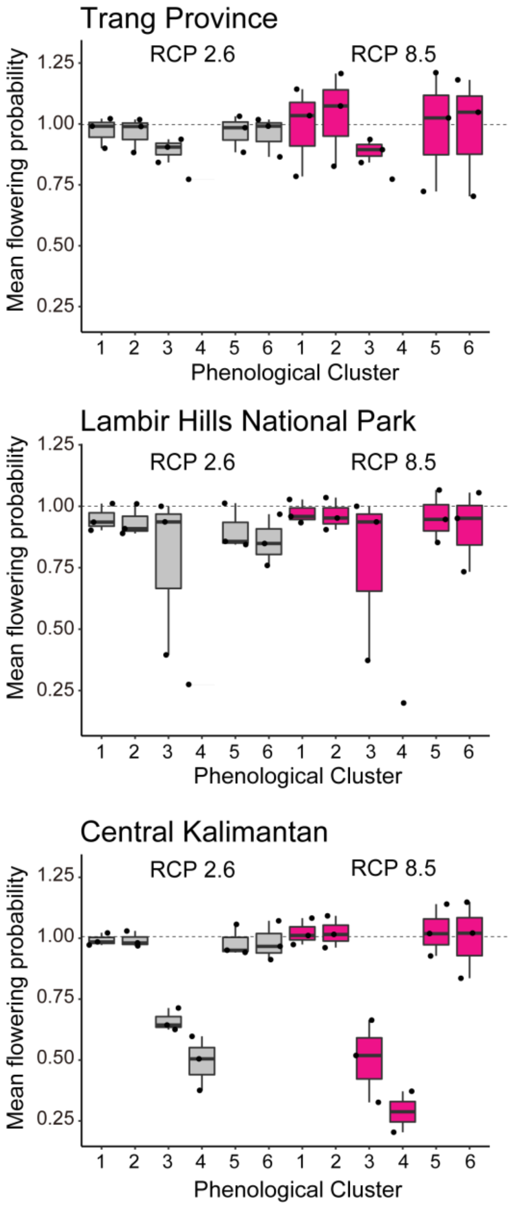

Supplementary Figure 6 | Predictions of future flowering phenology under two climate scenarios (RCP2.6 and RCP8.5) in three regions

The means ± standard errors of the normalized flowering probabilities predicted for 2050–2099 for each phenological cluster under two climate scenarios in three regions (Trang Province, Lambir Hills National Park, and central Kalimantan) are shown. The means and standard errors across three GCMs were calculated from predictions by GDFL–EMS2M, IPSL–CM5A-LR, and MIROC5. For each model and each phenological cluster, the prediction was normalized by the historical climate conditions during 1976–1996. A dotted line indicates the level that is equal to the one for 1976–1996 period. The horizontal line inside each box and the length of the box indicate the median and the interquartile range (the range between the 25th and 75th percentiles), respectively. The whiskers indicate points within 1.5 times the interquartile range. When the maximum flowering probability was less than 0.005 for both periods (1976–1996 and 2050-2099), we omitted the results because of the very low flowering probability.

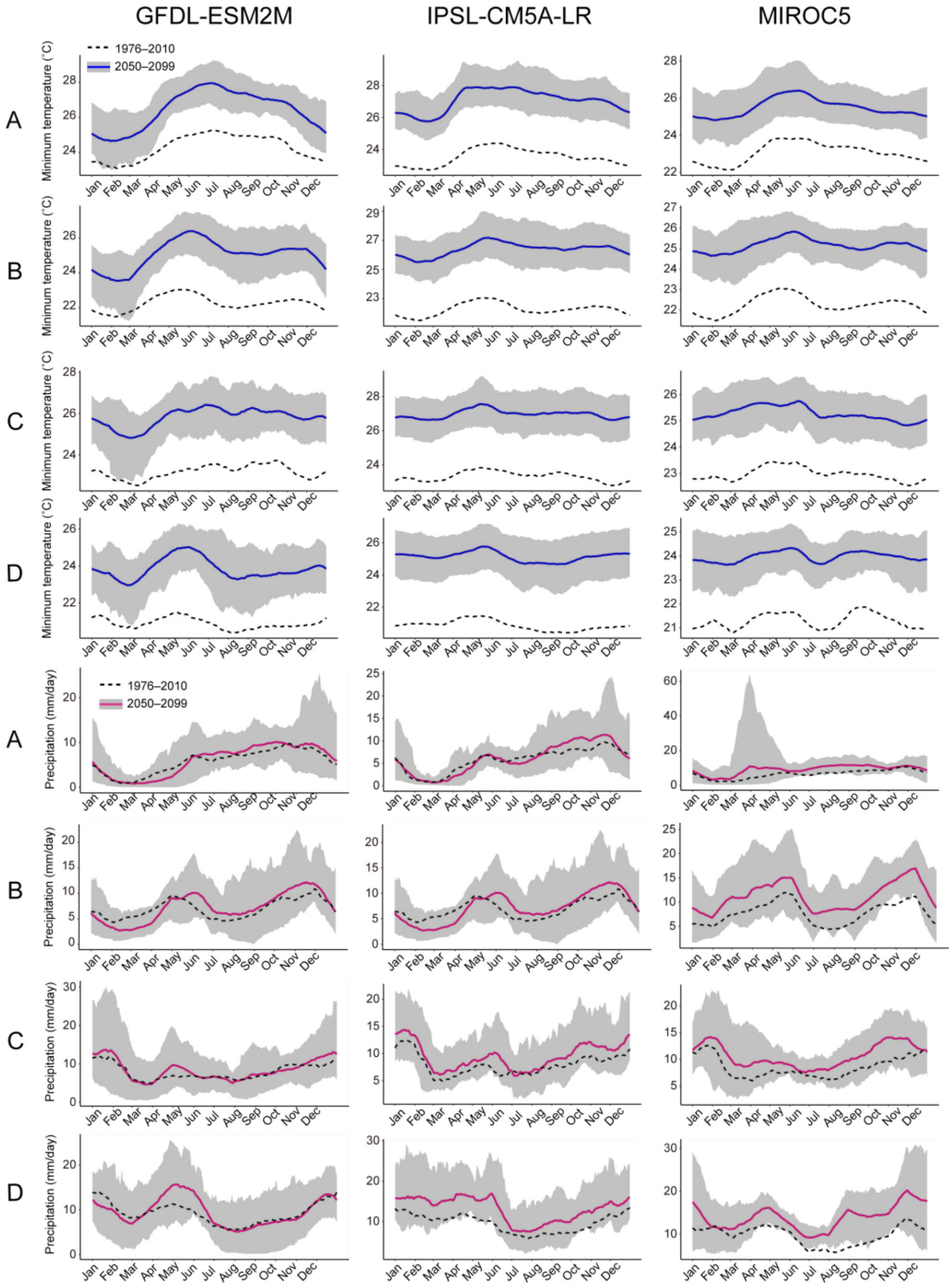

Supplementary Figure 7 | Seasonality in the future climate under the RCP8.5 scenario for each of three GCMs in four regions in Southeast Asia

Averages (blue line) ± standard errors (envelope) of the minimum temperature and averages (pink line) ± standard errors (envelope) of precipitation calculated from the 30-day running means during 2050–2099. Dotted lines indicate the average minimum temperature or precipitation value during the 1976–2010 period. A: Trang province, B: FRIM, C: Lambir Hills National Park, and D: central Kalimantan (see the map in Fig. 6a).

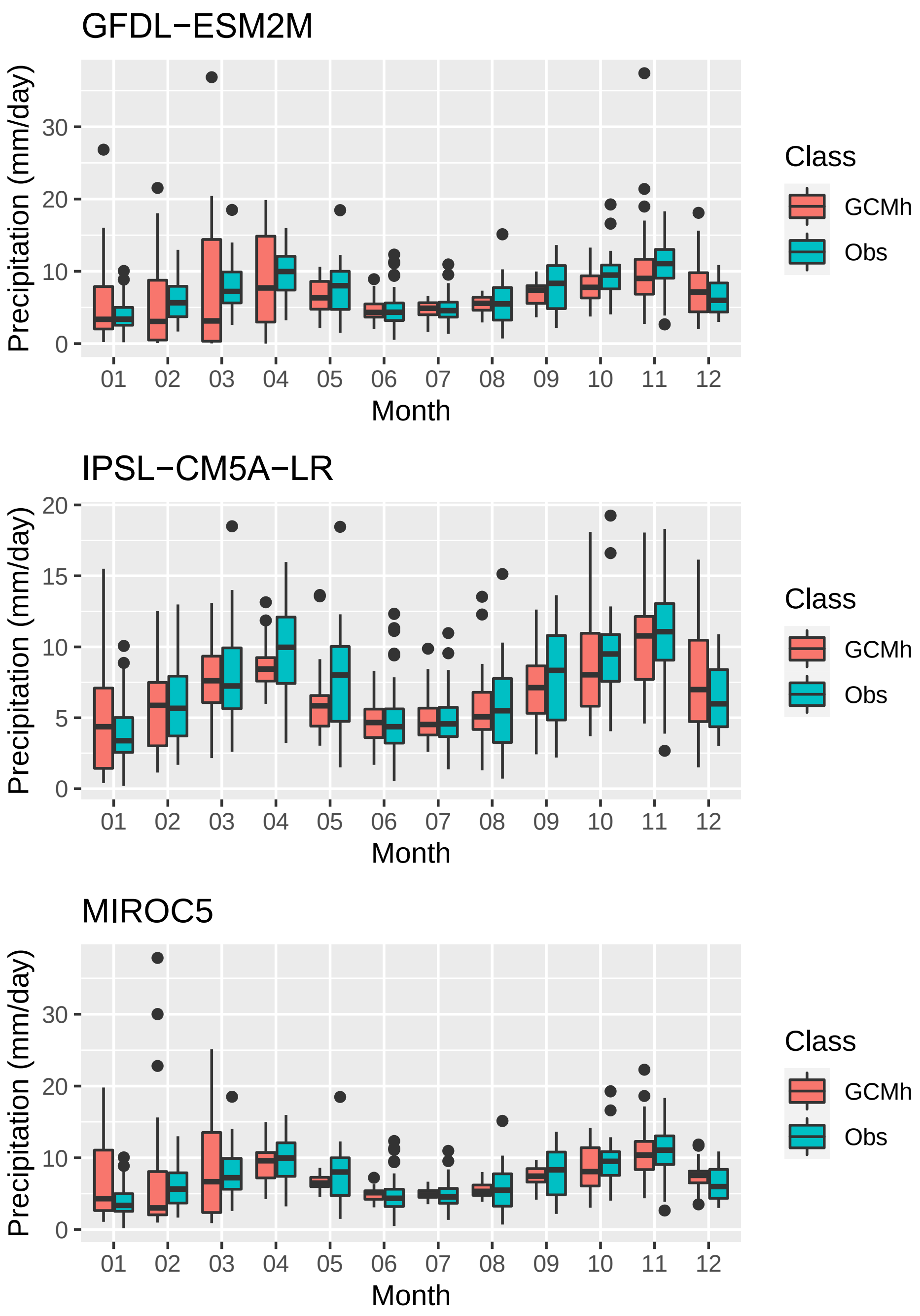

Supplementary Figure 8 | The comparison of the seasonal trend of the precipitation between the historical run of each GCM without bias correction and the observation data. The data used for the boxplot is during 1976–2004. The red boxes represent the data of GCMs and the green boxes represent the observation data. The upper and lower lines are 1st quartile - 1.5 x (3rd - 1st quartile) or 3rd quartile + 1.5 x (3rd quartile - 1st quartile).

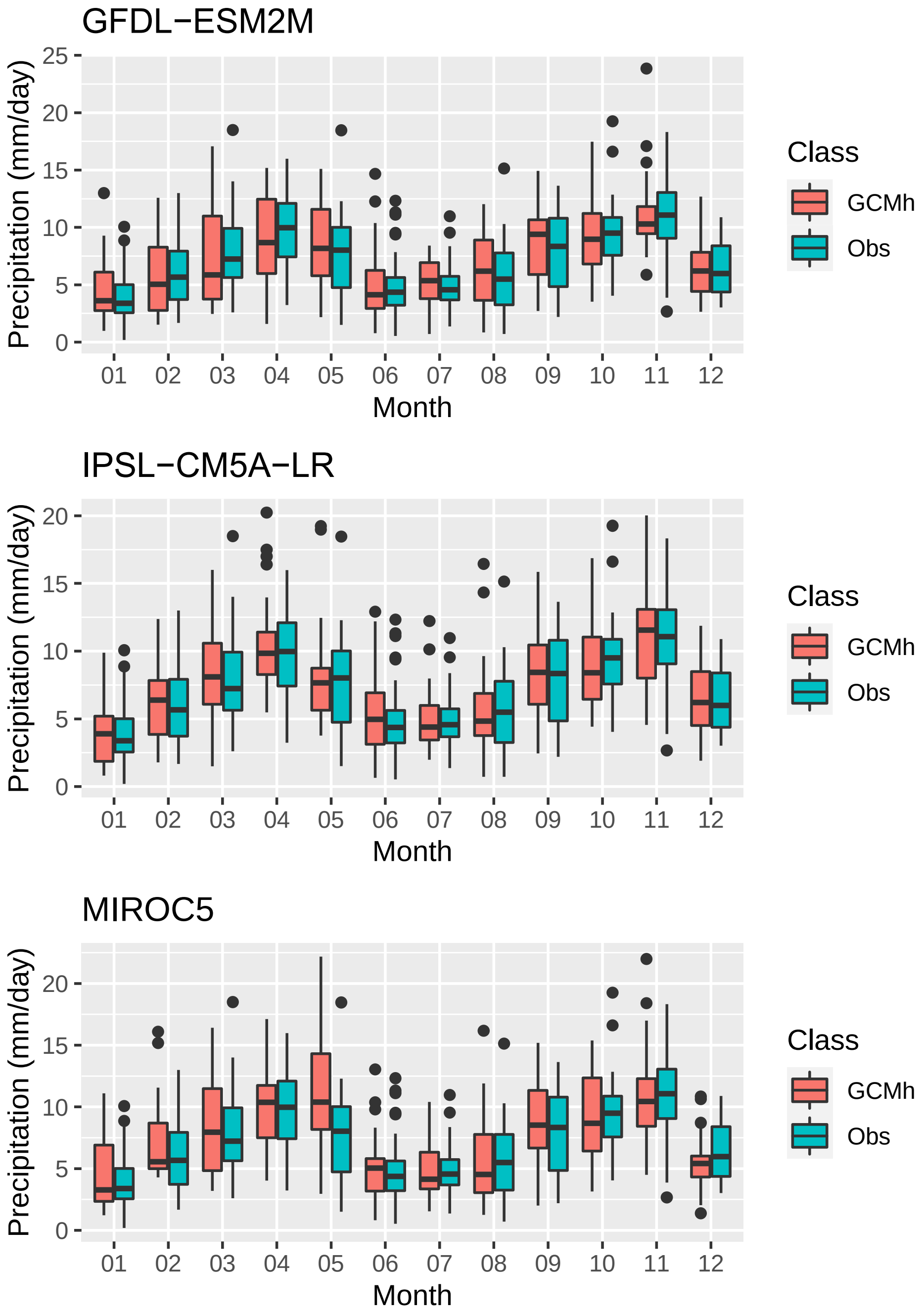

Supplementary Figure 9 | The comparison of the seasonal trend of the precipitation between the historical run of each GCM with bias correction and the observation data. The data used for the boxplot is during 1976–2004. The red boxes represent the data of GCMs and the green boxes represent the observation data. The upper and lower lines are 1st quartile - 1.5 x (3rd - 1st quartile) or 3rd quartile + 1.5 x (3rd quartile - 1st quartile).

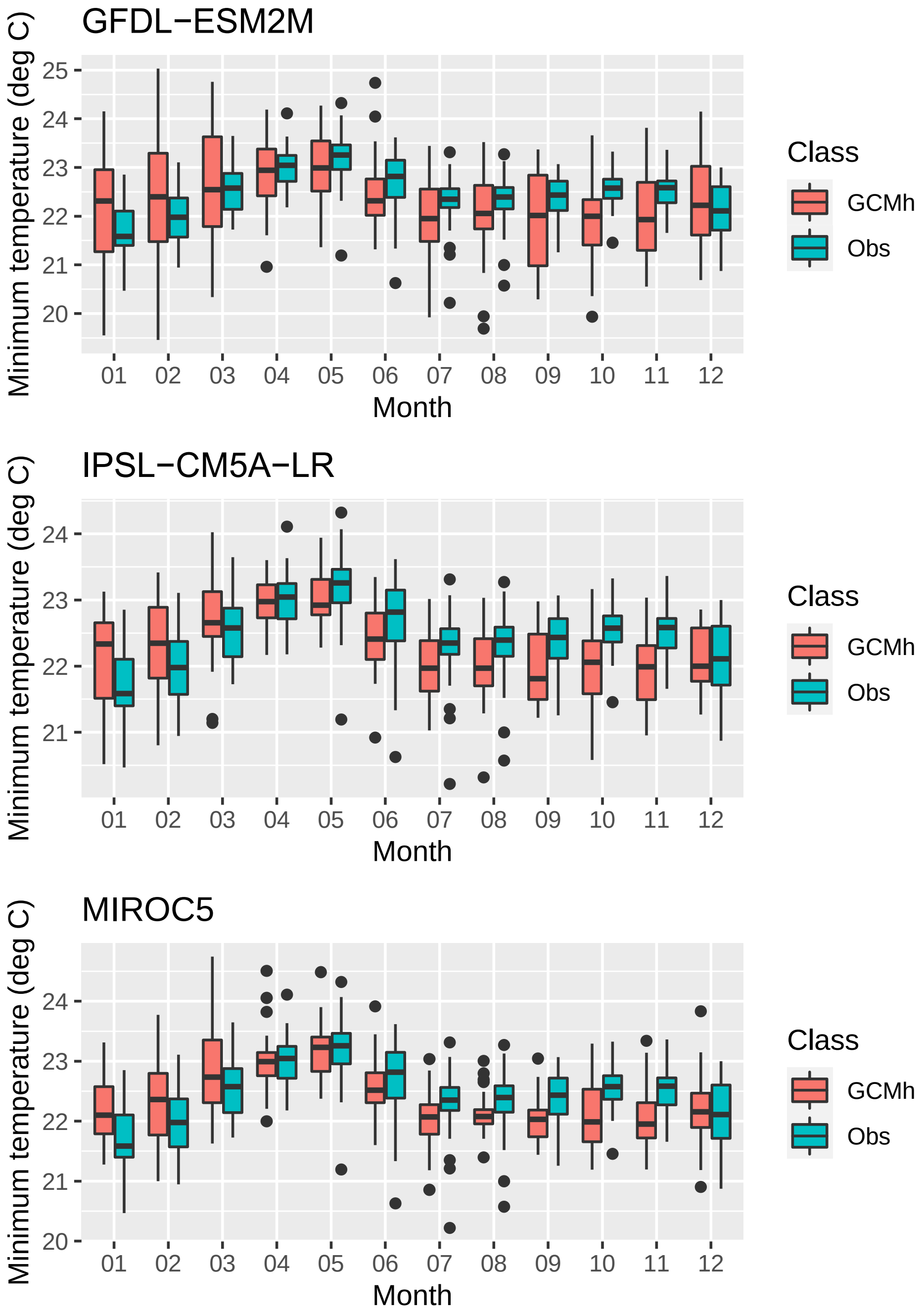

Supplementary Figure 10 | The comparison of the seasonal trend of the minimum temperature between the historical run of each GCM without bias correction and the observation data. The data used for the boxplot is during 1976–2004. The red boxes represent the data of GCMs and the green boxes represent the observation data. The upper and lower lines are 1st quartile - 1.5 x (3rd - 1st quartile) or 3rd quartile + 1.5 x (3rd quartile - 1st quartile).

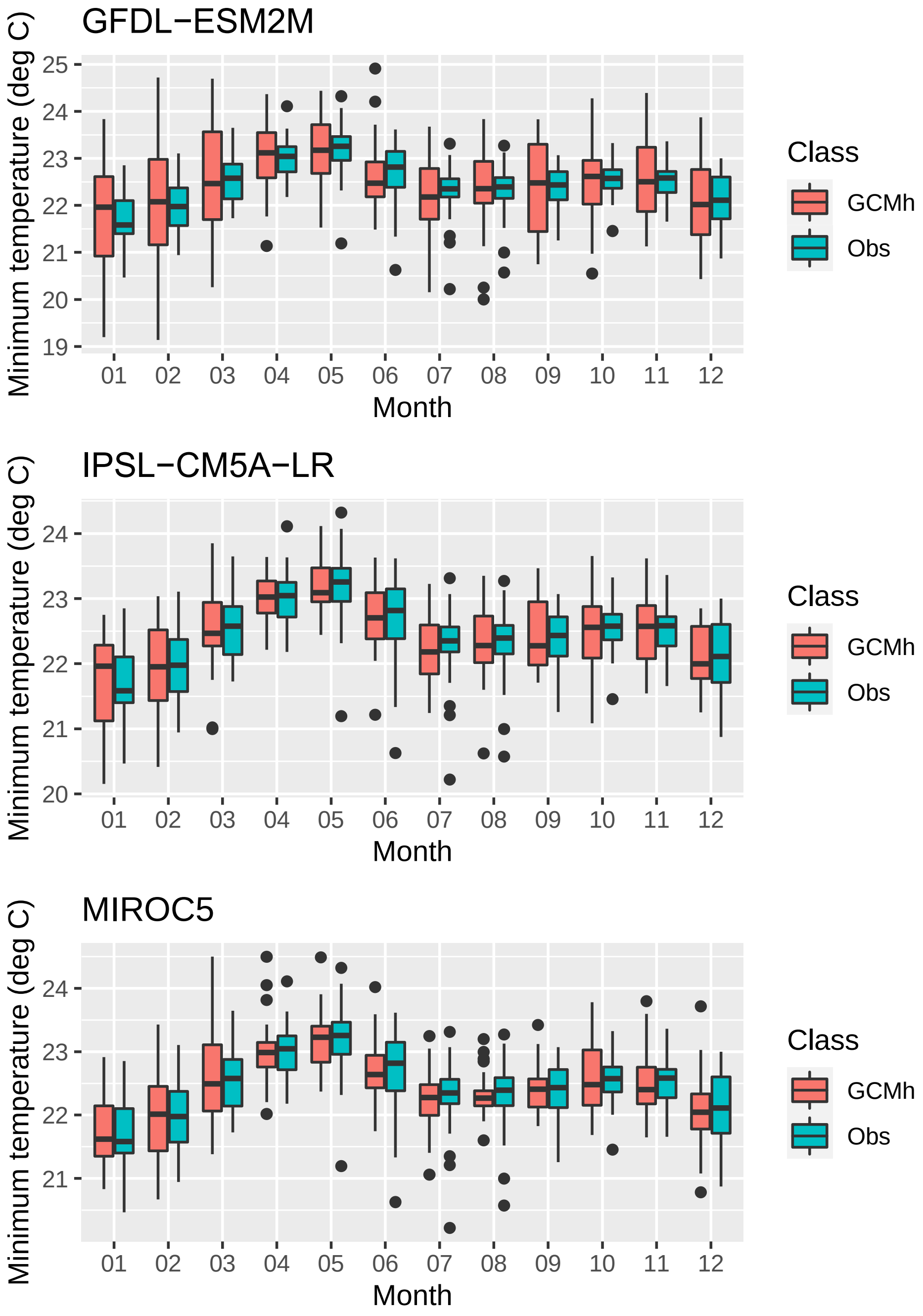

Supplementary Figure 11 | The comparison of the seasonal trend of the minimum temperature between the historical run of each GCM with bias correction and the observation data. The data used for the boxplot is during 1976–2004. The red boxes represent the data of GCMs and the green boxes represent the observation data. The upper and lower lines are 1st quartile - 1.5 x (3rd - 1st quartile) or 3rd quartile + 1.5 x (3rd quartile - 1st quartile).

Supplementary Table 1 | Results of the Mann–Kendall test for detecting long-term trends in the proportions of flowering and fruiting species. The mean change rate was estimated using linear regressions of the monthly flowering or fruiting data at the community level, including 210 tropical tree species.

Phenological trait	Mann–Kendall τ	*P* value	Mean change rate per decade	2.5% quantile	97.5% quantile
Flowering	−0.20	2.13E-03	−0.87E-02	−1.43E-02	−3.12E-03
Fruiting	−0.23	8.69E-12	−1.74E-02	−2.41E-02	−1.06E-02

Supplementary Table 2 | Parameters and fitting results of the best model. Flowering was assumed to be cued either by the drought unit only (DU) or by a synergism of cool temperatures and drought (CU × DU). In the table, α and β represent the coefficients of the intercepts and slopes of the logistic regression equations. The best models and parameter values are presented in bold. AUC-train indicates the area under the ROC curve (AUC) obtained using training data from June 1976 to March 1996. AUC-valid indicates the AUCs obtained using validation data from July 1997 to April 2005. AUC-valid could not be calculated for cluster 4 because no flowering events were predicted.

Phenological cluster	Flowering cue	n_1 (day)	n_2 (day)	$\bar{D}$ (mm)	$\bar{C}$ (°C)	α	β	AUC train	AUC valid
Cluster 1	DU	25	38	3.6	–	−2.75	1.01	0.64	0.62
Cluster 2	DU	27	41	4.1	–	−3.02	0.95	0.66	0.71
Cluster 3	CU × DU	26	44	4.4	20.3	−4.47	0.20	0.76	0.76
Cluster 4	CU × DU	33	19	4.8	19.4	−5.93	0.66	0.70	n.a.
Cluster 5	DU	38	29	3.7	–	−3.91	1.11	0.69	0.78
Cluster 6	DU	38	32	5.0	–	−3.86	0.93	0.78	0.79
