# Supplementary material for: Impacts of climate change on reproductive phenology in tropical rainforests of Southeast Asia
Source: Commun Biol. 2022 Apr 21;5:311. doi: 10.1038/s42003-022-03245-8 (PMC9023445; doi:10.1038/s42003-022-03245-8)
Supplement: Supplementary file 2 — Description for Additional Supplementary Materials [file 42003_2022_3245_MOESM2_ESM.pdf]

Description of Additional Supplementary Files

File name: Supplementary Data 1
Description: Species list

File name: Supplementary Data 2
Description: Order of species listed from the highest to lowest flowering frequency

File name: Supplementary Data 3
Description: Results of model fitting

File name: Supplementary Data 4
Description: List of dipterocarp species classified into six phenological clusters

File name: Supplementary Data 5
Description: Source code for DU model

File name: Supplementary Data 6
Description: Source code for CUDU model
