# Supplementary material for: Impacts of climate change on reproductive phenology in tropical rainforests of Southeast Asia
Source: Commun Biol. 2022 Apr 21;5:311. doi: 10.1038/s42003-022-03245-8 (PMC9023445; doi:10.1038/s42003-022-03245-8)
Supplement: Supplementary file 9 — Reporting Summary [file 42003_2022_3245_MOESM9_ESM.pdf]

nature portfolio

Corresponding author(s): Akiko Satake
Shinya Numata

Last updated by author(s): 28/01/2022

Reporting Summary

Nature Portfolio wishes to improve the reproducibility of the work that we publish. This form provides structure for consistency and transparency in reporting. For further information on Nature Portfolio policies, see our Editorial Policies and the Editorial Policy Checklist.

Statistics

For all statistical analyses, confirm that the following items are present in the figure legend, table legend, main text, or Methods section.

n/a	Confirmed	
☐	☒	The exact sample size (n) for each experimental group/condition, given as a discrete number and unit of measurement
☐	☒	A statement on whether measurements were taken from distinct samples or whether the same sample was measured repeatedly
☐	☒	The statistical test(s) used AND whether they are one- or two-sided<br>*Only common tests should be described solely by name; describe more complex techniques in the Methods section.*
☐	☒	A description of all covariates tested
☒	☐	A description of any assumptions or corrections, such as tests of normality and adjustment for multiple comparisons
☐	☒	A full description of the statistical parameters including central tendency (e.g. means) or other basic estimates (e.g. regression coefficient) AND variation (e.g. standard deviation) or associated estimates of uncertainty (e.g. confidence intervals)
☐	☒	For null hypothesis testing, the test statistic (e.g. F, t, r) with confidence intervals, effect sizes, degrees of freedom and P value noted<br>*Give P values as exact values whenever suitable.*
☒	☐	For Bayesian analysis, information on the choice of priors and Markov chain Monte Carlo settings
☐	☒	For hierarchical and complex designs, identification of the appropriate level for tests and full reporting of outcomes
☐	☒	Estimates of effect sizes (e.g. Cohen's d, Pearson's r), indicating how they were calculated

Our web collection on statistics for biologists contains articles on many of the points above.

Software and code

Policy information about availability of computer code

Data collection	*Provide a description of all commercial, open source and custom code used to collect the data in this study, specifying the version used OR state that no software was used.*
Data analysis	The codes used for model fitting are provided as Source Data.

For manuscripts utilizing custom algorithms or software that are central to the research but not yet described in published literature, software must be made available to editors and reviewers. We strongly encourage code deposition in a community repository (e.g. GitHub). See the Nature Portfolio guidelines for submitting code & software for further information.

Data

Policy information about availability of data

All manuscripts must include a data availability statement. This statement should provide the following information, where applicable:

- Accession codes, unique identifiers, or web links for publicly available datasets
- A description of any restrictions on data availability
- For clinical datasets or third party data, please ensure that the statement adheres to our policy

The phenology data of dipterocarp species used in this study are provided in the Extended Data 4. The meteorological data provided by the Malaysian Meteorological Department may not be resold, distributed or disclosed to third parties, as regulated by the Malaysian Meteorological Department. Other data that support the findings of this study are available from the corresponding authors (SN and AS) upon reasonable request.

Field-specific reporting

Please select the one below that is the best fit for your research. If you are not sure, read the appropriate sections before making your selection.

☐ Life sciences ☐ Behavioural & social sciences ☒ Ecological, evolutionary & environmental sciences

For a reference copy of the document with all sections, see nature.com/documents/nr-reporting-summary-flat.pdf

Ecological, evolutionary & environmental sciences study design

All studies must disclose on these points even when the disclosure is negative.

Study description

Monthly reproductive phenology data were collected from the “Bulletin Fenologi Biji Benih dan Anak Benih (Bulletin of Seed and Seedling Phenology),” which was deposited at the library of the Forest Research Institute Malaysia (FRIM). The bulletin reported seed and seedling availabilities and the flowering and fruiting phenology of trees at several research stations in Malaysia. The present study collected flowering and fruiting records of trees grown in FRIM arboretums located approximately 12 km northwest of Kuala Lumpur, Malaysia (latitude 3°24 'N, longitude 101°63 'E, elevation 80 m).

Research sample

There are both dipterocarp and non-dipterocarp arboretums in FRIM, both of which were founded in 1929. These arboretums preserve and maintain living trees for research and other purposes. Because only one or two individuals per species are grown at the FRIM arboretums, the flowering and fruiting phenology were monitored using these individuals.

Sampling strategy

NA

Data collection

Each month, three research staff members of FRIM with sufficient phenology monitoring training made observations with binoculars to record the presence of flowers and fruits on trees of each species on the forest floor from April 1976 to September 2010. The phenological status of the trees was recorded as "flowering" during the developmental stages from flower budding to blooming and as "fruiting" during the developmental stages from the occurrence of immature fruit to fruit ripening.

Timing and spatial scale

Monthly reproductive phenology data were recorded over 35 years (from April 1976 to September 2010) at FRIM.

Data exclusions

The original data included 112 dipterocarp and 240 non-dipterocarp species. We excluded 17 dipterocarp and 125 non-dipterocarp species based on the following five criteria for data accuracy.

1) Percentage of missing values is less than or equal to 50%: If the monthly flowering or fruiting phenology data of a species included a substantially large number of missing values (more than 50%), the species was excluded.
2) Stable flowering period: We considered an observation to be unreliable if the flowering period was significantly different among flowering events (if the coefficient of variation in the flowering period was larger or equal to 1.0).
3) Flowering period is shorter than or equal to 12 months: We considered an observation to be unreliable if the flowering period was longer than 12 months because it was unlikely that the same tree would flower continuously for longer than 1 year.
4) The flowering and fruiting frequencies were not significantly different between the first and second half of the census period: When the flowering frequency was zero for the first half of the observation period but was larger than 0.1 for the second half of the observation period, or when the flowering frequency was zero for the second half of the observation period but was larger than 0.1 for the first half of the observation period, we removed these species because data are not reliable (e.g. physiological conditions may have changed significantly). We adopted the same criteria for the fruiting phenology data.
5) We removed overlapping species, herb species, and specimens with unknown species names.

After removing unreliable species based on the five criteria explained above, we obtained 95 dipterocarp and 115 non-dipterocarp species (Extended Data 1). We used these species for further analyses.
The original data included 112 dipterocarp and 240 non-dipterocarp species. We excluded 17 dipterocarp and 125 non-dipterocarp species based on the following five criteria for data accuracy.

1) Percentage of missing values is less than or equal to 50%: If the monthly flowering or fruiting phenology data of a species included a substantially large number of missing values (more than 50%), the species was excluded.
2) Stable flowering period: We considered an observation to be unreliable if the flowering period was significantly different among flowering events (if the coefficient of variation in the flowering period was larger or equal to 1.0).
3) Flowering period is shorter than or equal to 12 months: We considered an observation to be unreliable if the flowering period was longer than 12 months because it was unlikely that the same tree would flower continuously for longer than 1 year.
4) The flowering and fruiting frequencies were not significantly different between the first and second half of the census period: When the flowering frequency was zero for the first half of the observation period but was larger than 0.1 for the second half of the observation period, or when the flowering frequency was zero for the second half of the observation period but was larger than 0.1 for the first half of the observation period, we removed these species because data are not reliable (e.g. physiological conditions may have changed significantly). We adopted the same criteria for the fruiting phenology data.
5) We removed overlapping species, herb species, and specimens with unknown species names.

After removing unreliable species based on the five criteria explained above, we obtained 95 dipterocarp and 115 non-dipterocarp species (Extended Data 1). We used these species for further analyses.

Reproducibility	NA
Randomization	NA
Blinding	Three different research staff members of FRIM with sufficient phenology monitoring training made observations.

Did the study involve field work? ☒ Yes ☐ No

Field work, collection and transport

Field conditions	natural conditions
Location	the Forest Research Institute Malaysia (FRIM)
Access & import/export	FRIM arboretums located approximately 12 km northwest of Kuala Lumpur, Malaysia (latitude 3°24 'N, longitude 101°63 'E, elevation 80 m).
Disturbance	NA

Reporting for specific materials, systems and methods

We require information from authors about some types of materials, experimental systems and methods used in many studies. Here, indicate whether each material, system or method listed is relevant to your study. If you are not sure if a list item applies to your research, read the appropriate section before selecting a response.

Materials & experimental systems

n/a	Involved in the study	
☒	☐	Antibodies
☒	☐	Eukaryotic cell lines
☒	☐	Palaeontology and archaeology
☒	☐	Animals and other organisms
☒	☐	Human research participants
☒	☐	Clinical data
☒	☐	Dual use research of concern

Methods

n/a	Involved in the study	
☒	☐	ChIP-seq
☒	☐	Flow cytometry
☒	☐	MRI-based neuroimaging
